# A participatory approach to health promotion for informal sector workers in Thailand

**DOI:** 10.5249/jivr.v2i2.36

**Published:** 2010-06

**Authors:** Aniruth Manothum, Jittra Rukijkanpanich

**Affiliations:** ^a^Faculty of Engineering, Chulalongkorn University, Thailand.

## Abstract

**Background:**

This study aims to promote occupational health in the informal sector in Thailand by using a participatory approach. The success of the intervention is based on an evaluation of the informal sector workers, a) knowledge, attitudes, and behaviors in occupational health and safety, b) work practice improvement, and c) working condition improvement.

**Methods:**

This study applies the Participatory Action Research (PAR) method. The participants of the study consisted of four local occupations in different regions of Thailand, including a ceramic making group in the North, a plastic weaving group in the Central region, a blanket making group in the Northeast, and a pandanus weaving group in the South. Data was collected using both qualitative and quantitative methods through questionnaires, industrial hygiene instruments, and group discussions.

**Results:**

The results showed that the working conditions of the informal sector were improved to meet necessary standards after completing the participatory process. Also, the post-test average scores on 1) the occupational health and safety knowledge, attitudes and behaviors measures and 2) the work practice improvement measures were significantly higher than the pre-test average scores (P less than 0.05).

**Conclusions:**

The results demonstrate that the participatory approach is an effective tool to use when promoting the health safety of the informal sector and when encouraging the workers to voluntarily improve the quality of their own lives.

## Introduction

The informal sector plays an important role in economic development by creating employment. Studies of the informal sector workers from many countries around the word, particularly in the developing countries, find that there has been a rapidly increasing number of informal sector workers.^1,2^

According to Phoon, the “informal sector” is understood to imply the use of labor-intensive working methods, with most of the workforce being self-employed. In general, these workers have no permanent employment status. They are not covered by the government’s legal and welfare systems. Moreover, they are not protected by an occupational health and safety laws and regulations.^3^ The International Labor Organization (ILO) introduced the concept of the informal sector. They described the informal sector as consisting of a very small scale units producing and distributing goods and services. The informal sector is composed largely of independent self-employed producers in urban and rural areas of the developing countries. Some of the units also employ family labor and/or a few hired workers or apprentices.^4^ Informal sector makes a significant contribution to economic activity, accounting for an estimated 30 to 60% of the gross national product in many less developed countries.^3^ In Thailand, more than 24.1 million people are working in the informal sector. Regions with the greatest number of informal sector workers are the Northeast region with 10.1 million (41.9%) followed by the North region with 5.2 million (21.5%). The smallest numbers of informal-sector workers are sited in the South region with 3.1 million (12.8%).^5^ These workers have played an important role in strengthening the economy of the countries. It was estimated that, in 2005, the informal sector generated income of 2,000,000 million baht a year, or 43% of Thailand’s gross domestic product.^5,6^

However, the workers in the informal sector are encountering various problems affecting the economic growth of their countries. One of the major problems is that informal sector workers are often provided with unsuitable occupational health and safety (OHS) workplaces. They often work in poor, sub-standard working conditions and are exposed to various hazards without proper knowledge concerning the use of personal protective equipment (PPE). Moreover, informal sector workers lack necessary health information.^7^

Among the 24.1 millions informal sector workers in Thailand, 4.2 million of them have experienced injuries or accidents. The majority (2.8 million) of these injured workers were cut or wounded by sharp materials. Five hundred and twenty nine thousand had fallen from heights. Three hundred and thirty two thousand were injured from being hit by machineries or tools in their workplaces. One hundred and ninety five thousand of were injured in automobile accidents. Sixty five thousand informal sector workers had been exposed to chemical substances.^5^In Thailand, formal sector workers who are injured at work have access to compensation under the 1994 Workmen’s Compensation Act.^8^ However, informal sector workers are not protected under this law.

The International Labor Organization (ILO) showed that informal sector workers in industrially developing countries are exposed to poor working environments, low safety and health standards, and environmental hazards. Such exposure impairs their health and productivity as well as the general well-being and quality of life of the informal workers and their families. But often they are not even aware of the risks they face and, if they are, they do not know how to avoid them. Low levels of technology, low productivity, irregular employment relationships and improper investment capacity tend to increase the exposure of the informal workers to occupational accidents and diseases. Inadequate technical and managerial skills are also aggravating factors.^1^

There are several ways to solve OHS problems, for example, providing education and training, enforcing existing laws, and/or promoting participatory activities.^9-10^ However, the economic and cultural contexts of Thai informal-sector workers favor using a participatory approach. A participatory approach promotes the participation of the stakeholders in the decision making and problem solving process through the use of the participatory process. The strength of the participatory approach consists of its focus on economical and achievable management goals, effective problem solving methods, and community based approaches.The participatory approach helps motivating workers to participate in the problem solving process.^2,11^

The specific aim of this study towards this goal is to evaluate the participatory approach based on the informal sector workers’ a) knowledge, attitudes, and behaviors in OHS, b) work practice improvement, and c) working condition improvement.

## Methods

This study used the participatory action research (PAR) method because the core concept of this study focused on the participation of the informal sector workers in sharing ideas and synthesizing information using the workers’ own local wisdom.

**Sample selection**

A purposive sampling technique was applied in the study. In selecting the sample size, a walk-through survey was conducted in the target areas. This process was aimed at identifying the career groups most at risk by using five criteria: 1) the career group involved at micro enterprise, 2) encountered high risk at work, 3) work period continuity was, at least, one year, 4) the number of members in the group were between twenty to fifty, and 5) the extend to which the career was popular and distinctive in the community.

Participants in the study were informal sector workers in micro-enterprises from four regions of Thailand—which consisted of 24 ceramic workers from Lampang province in the North region, 23 plastic weavers from Ang Thong province in the Central region, 22 blanket workers from Nakhon Ratchasima province in the Northeast region, and 20 pandanus weavers from Trang province in the South region.

**The implementation of a participatory process**

The stakeholders involved in the OHS problems include local government officials, non-governmental organizations (NGOs), and the leaders of the informal sector workers. All these entities are responsible for solving the OHS problems. However, prior to implementing the participatory process, they lacked the potential to provide any systematic OHS management including: 1) lack of corrective and preventive mechanisms in the governmental sector—for example, there were no appropriate guidelines concerning specific authorized functions and 2) lack of potential to create appropriate tools and procedure to solve OHS problems in local organizations such as the Provincial offices of labor, Health offices, and Sub-district administration organizations.

In general, the stakeholders in informal sectors, especially the workers, have insufficient concepts and impractical guidelines concerning how to handle OHS problems appropriately. Therefore, the development of a participatory process is extremely necessary for preventing future problems and decreasing the health affects caused by work hazards in the informal sector. Additionally, a participatory process can lead to sustainable and holistic problem solving.

According to the reasons presented above, the use of a participatory approach in the current study is justified. A participatory approach is considered an effective strategy in solving OHS problems and seems appropriate for use with informal sector workers.^11^ During the process of model development, steps were taken to motivate the workers to participate as much as possible. Also, in the process of model development, the researchers placed a high value on the community’s culture in each target area.

**Figure 1: Concept for promotion of OHS management in the informal sector by using the participatory approach:**
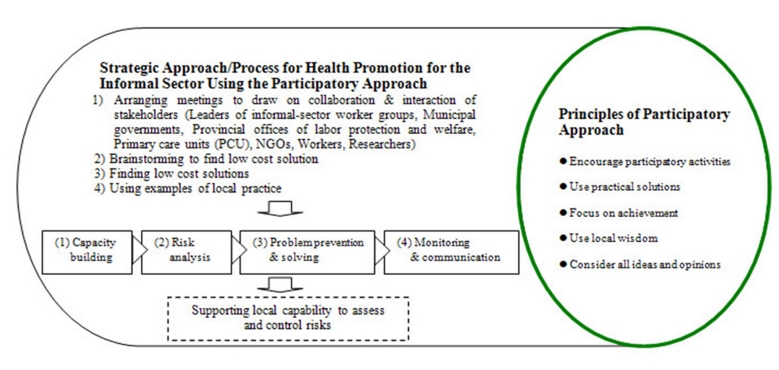


This study applied the concept of a participatory approach. This process of promoting OHS management was developed with the cooperation and participation of local networks. Local network participation included collaboration and interaction between stakeholders. All steps of the participatory process aim at increasing the workers’ self development related to health and safety—which corresponds to the dynamic model of Minkler^12^ (see Figure 1 ).

The participatory process includes four processes as follows: 1) capacity building; its objective is to help informal sector workers learn concepts for improving work conditions, 2) risk analysis; its objective is to facilitate informal sector workers’ understanding of the risk factors associated with their occupations, 3) problem prevention and solving; its objective is to engage the informal sector workers in improving working conditions, and 4) monitoring and communication; its objective is to confirm benefits and sustain the problem solving activities.

The study groups participated in learning, analyzing, and solving the problems related to OHS by themselves, with appropriate guidance from the researchers. The details of each process were as follow:

(1)Capacity building: this process involved helping informal sector workers learn and understand their OHS problems by participatory learning activities such as group discussions on workshop tools and the best practices of other groups.

(2) Risk analysis: this process involved analyzing current/existing problems by applying the techniques of job safety analysis and walk-through surveys using the illustrated checklist to survey one’s own workplace. Risk assessment analysis was done to enable the workers to participate in identifying and assessing the risks associated with their own occupations.

(3) Problem prevention and solving: this process followed the risk assessment analysis. The solutions offered by the informal sector workers were topics of the group discussions that utilized the Work Improvement in Small Enterprises (WISE) techniques. The WISE technique was proposed by the International Labor Office and it helps solutions incorporate local folk wisdom to assure that the solutions are practical, simple, economical, and appropriate for the occupational groups.^13^The solution plans were recorded in the improvement plan forms after all of the members of the sample groups agreed upon them.

(4) Monitoring and communication: this process was used to follow up the former processes. The researchers monitored by comparing the plans with actual practices. Included in this process, were discussions with and feedback from the stakeholders. When any problems were presented, the stakeholders participated in solving the problems. Therefore, the monitoring and communication process, which included following up, receiving feedback, and solving problems jointly, was a two-way communication process which led to a better understanding of the improvement of OHS conditions based on community practices.

**Measuring knowledge, attitudes and behaviors**

The measurement of the informal sector workers’ knowledge, attitudes and behaviors involved using the questionnaire proposed by the Department of Labor Protection and Welfare, Ministry of Labor, Thailand.^14^This questionnaire consists of three parts:

1)Measuring the participants’ knowledge of OHS including their understanding of the definition of accidents, causes of accidents, prevention of hazards and dangers, and the use of PPE. Questions with multiple response categories (i.e. multiple choices) were used and respondents were instructed to select only one answer. The guideline for the scoring was to give 1 point for the correct answer and 0 for giving a wrong answer. The participants’ level of knowledge was assessed using the following scoring scheme: low level of knowledge = scoring less than 50% of the total scores or scoring 0 - 2.5 points; medium level of knowledge = scoring equal to 50-75% of the total score or > 2.5 - 3.75 points; and high level of knowledge = scoring higher than 75% of the total score or > 3.75-5 points.

2)Measuring participants’ attitudes toward OHS. Likert scale type questions were used to measure attitudes toward five aspects of OHS using both positive and negative phrases—i.e. attitudes about the cause and prevention of accidents, the use of PPE, etc. The following scoring scheme was used to group participants into various levels of attitudes toward OHS: negative attitude toward safety = scoring less than 50 % of the total score or 0 – 2.5 points; indifferent attitude toward safety = scoring 50-75% of the total score or > 2.5 – 3.75 points; and positive attitude toward safety = scoring higher than 75% of the total score or > 3.75 - 5 points.

3)Measuring participants’ OHS-safety related behavior using questions with the “yes-no” response categories. The questions covered a range of topics—including if participants had any experience in using various tools, their knowledge regarding work safety, and the condition of the tools, etc. The condition of the tools was referred to the physical condition such as being ready to be used, being broken or damaged. Participants received 1 point for report of a safe behavior and 0 for the report of an unsafe behavior. The following scoring scheme was used to group participants into various levels of OHS-related safety behavior: low level of safe behavior = scoring less than 50 % of the total score or 0 – 2.5 points; medium level of safe behavior = scoring 50-75% of the total score or > 2.5 – 3.75 points; and high level of safe behavior = scoring higher than 75% of the total score or > 3.75 - 5 points.

**Measuring work practice improvement**

The observation of work practice improvement involved using a checklist proposed by the Work Improvement Small Enterprises (WISE) and Work Improvement for Safe Homes (WISH) programs developed by the ILO.^13^ Both the WISE and WISH methods focus on the participatory approach—convincing the owners of small and medium sized enterprises (SMEs) and informal sector groups to join in the self-help practice of solving OHS problems.

The checklist consists of the 7 practice improvement sections including improvements in the: materials storage and handling, work-station design, machine safety, control of chemicals, working conditions, welfare facilities and premises—with a total score of 120 points. Levels of the “work practice improvement” were assessed using the following classification scheme: low level of work practice improvement = scoring less than 50 % of the total score, or 0 - 60 points; medium level of work practice improvement = scoring equal to 50-75% of the total score or > 60 - 90 points; and high level of work practice improvement = scoring higher than 75% of the total score or > 90 - 120 points

**Measuring working conditions improvement**

The selection of priorities to improve the working conditions for the informal sector workers were determined by the group discussions which included analyzing risks involved in the work processes, identifying occupational health hazards at the workplace, and brain-storming to find the best solutions for the work-related problems. Participation in the group discussions varied in each organization depending on the culture of the organization and the type of occupation. Nevertheless, the mechanism of the participatory process allowed the many participants to share their opinions in determining and solving their priority issues.

The industrial hygiene instruments was used to measure; 1) the “heat condition”, using a heat measurement for the ceramic making group using a WBGT heat stress monitor with the WBGT index that utilizes ISO 7243-1982 and, 2) the “light condition” for the plastic weaving, blanket making, and pandanus weaving groups using a lux meter (EA30, Extech Instruments Co., Massachusetts, USA). The selected samples were analyzed by; 1) the Regional Office for the Improvement of Working Conditions and Environment, Department of Labor Protection and Welfare, Ministry of Labor, Thailand, and 2) the Faculty of Science, Phuket Rajabhat University.

**Data analysis**

Pre and post-test data were collected and compared to provide descriptive statistics (percentages, means and standard deviations) and inferential statistics (paired t-tests).

## Results

The results are divided into four sections: 1) participants’ characteristics, 2) participants’ knowledge, attitudes and behaviors in OHS, 3) work practice improvements, and 4) working condition improvements.

**Participants’ characteristics**

Table 1 presents the characteristics of the studied participants. A total of 89 informal sector workers from four regions of the Thailand were included in the study.

**Table:** Table 1. **Population characteristics of the informal sector workers**

	Ceramic making(n=24)	Plastic weaving(n=23)	Blanket making(n=22)	Pandanus weaving(n=20)
Gender				
Male	5 (20.8%)	2 (8.7%)	5 (22.7%)	2 (10.0%)
Female	19 (79.2%)	21 (91.3%)	17 (77.3%)	18 (90.0%)
Age				
<19	1 (4.2%)	0	1 (4.5%)	0
20-29	4 (16.6%)	5 (21.7%)	2 (9.1%)	1 (5.0%)
30-39	10 (41.7%)	10 (43.5%)	8 (36.4%)	6 (30.0%)
40-49	9 (40.9%)	4 (17.4%)	7 (31.8%)	9 (45.0%)
50-59	2 (8.3%)	4 (17.4%)	4 (18.2%)	1 (5.0%)
>60	0	0	0	0
Marital status				
Single	9 (37.5%)	10 (43.5%)	3 (13.6%)	5 (25.0%)
Married	14 (58.3%)	11 (47.8%)	16 (72.7%)	15 (75.0%)
Divorced	1 (4.2%)	2 (8.7%)	3 (13.6%)	0
Experience				
<1	5 (20.8%)	3 (13.0%)	1 (4.5%)	0
1-4	9 (37.5%)	12 (52.2%)	7 (31.8%)	3 (15.0%)
5-7	5 (20.8%)	3 (13.0%)	10 (45.5%)	2 (10.0%)
7-10	3 (12.5%)	3 (13.0%)	1 (4.5%)	1 (5.0%)
>10	2 (8.3%)	2 (8.7%)	3 (13.6%)	14 (70.0%)
Working hours/day (hours)				
<8	0	0	0	0
8	12 (50.0%)	12 (52.2%)	18 (81.8%)	15 (75.0%)
>8	12 (50.0%)	11 (47.8%)	4 (18.2%)	5 (25.0%)

Table 1 shows that the larger percentage of the informal sector workers in our study were women; ceramic workers (79.2%), plastic weavers (91.3%), blanket workers (77.3%), and pandanus weavers (90%). The highest age group was found in pandanus weaving sector with 15% of the worker being >60 years. The minimum age group was mostly found in blanket making with 4.5% being <19 years, followed by 4.2% in ceramic making sector. Most of participants in each of the sectors were married and 8.3, 8.7, 13.6 and 70% of ceramic making, plastic weaving, blanket making, and pandanus weaving workers had more than 10 years of work experience, respectively. Most of the workers worked 8 hours per day. However, 47.8% of plastic weavers, 18.2% of blanket workers, 25% of pandanus weavers work more than 8 hours.

**OHS knowledge, attitudes, and behaviors**

Table 2shows that, the participants’ OHS knowledge, attitudes, and behaviors scores across all sectors increased in the post-test (12.42, 12.57, 12.47, and 12.59 points respectively) and the difference between the pre and post-test average scores on the OHS knowledge, attitudes and behaviors measurements were statistically significantly (p < 0.05).

**Table:** Table 2: **Comparison of the mean scores of the informal sector workers’ OHS knowledge, attitudes, and behaviors before and after applying the participatory process**

Informal sector workers		Mean(SD) Knowledge attitudes, and behaviors	p-value
Ceramic making	Before	6.67 (0.91)	0.000
	After	12.42 (0.75)	
Plastic weaving	Before	5.31 (0.98)	0.000
	After	12.57 (0.76)	
Blanket making	Before	7.20 (0.82)	0.000
	After	12.47 (0.72)	
Pandanus weaving	Before	6.66 (0.86)	0.000
	After	12.59 (0.80)	

Paired t-test application, p<0.05 (Total possible scores for OHS knowledge, attitudes, and behaviors = 15 points)

**Work practice improvement**

Table 3 shows that, the work practice improvement means in all groups increased (106, 105.75, 117, and 118.50, respectively). As presented in Table 3, participants’ post-test mean scores on the work practice improvement measurements were also significantly higher than their pre-test average scores (p <0.05).

**Table:** Table 3: **Comparison of the mean scores of the informal sector workers’ work practice improvement before and after applying the participatory process**

Informal sector workers		Mean(SD) Work practice improvement	p-value
Ceramic making	Before	57.75 (2.21)	0.000
	After	106.00 (2.16)	
Plastic weaving	Before	58.00 (2.44)	0.000
	After	105.75 (2.50)	
Blanket making	Before	49.50 (1.29)	0.000
	After	117.00 (0.81)	
Pandanus weaving	Before	52.75 (1.25)	0.000
	After	118.50 (0.57)	

Paired t-test application, p<0.05 (Total possible scores for work practice improvement = 120 points)

**Working conditions**

During the participatory process, the workers and the stakeholders brainstormed and discussed possible solutions to their work-related problems. They suggested improvements tips for each work group, using local wisdom and the WISE technique including installing heat barriers for the ceramic making group, and increasing the lighting condition for the plastic weaving group, blanket making group, and pandanus weaving group.

High temperature working condition is considered a serious problem for the ceramic workers-especially for those working near a ceramic furnace. Thus, the stakeholders introduced heat barriers between the furnaces and the workers. Using information shared in the group discussions, heat barriers were designed and made by the local workers. The wet bulb, dry bulb, and globe temperatures (WBGT) were measured to estimate the Heat Stress Indexes at the workplace. The results demonstrated that the introduction of heat barriers helped to protect the workers from the heat radiation caused by the furnace, due to a decreased in the rates of WBGT (see Table 4).

**Table:** Table 4. **Results of working conditions assessments in the workplace of the informal sector groups before and after improvement**

Informal sector workers	Working conditions	Process	Before Mean (SD)	After Mean (SD)	Standard
Ceramic making	Thermal condition(C°)	1) Dry bulb(n=3)	29.8 (0.057)	26.7 (0.100)	-
		2) Dry bulb(n=3)	35.2 (0.057)	30.8 (0.100)	-
		3) Glove(n=3)	43.1 (0.321)	32.3 (0.152)	-
		4) WBGT Index(n=3)	33.3 (0.057)	28.0 (0.288)	-
Plastic weaving	Lighting (lux) (lux)	1) Plastic cutting(n=3)	742 (3.214)	905 (0.587)	400*
		2) Plastic weaving(n=3)	750 (0.577)	915 (1.000)	800*
Blanket making	Lighting (lux) (lux)	1) Cloth cutting(n=3)	450 (4.041)	905 (0.577)	400*
		2) Putting stuff material(n=3)	400 (1.527)	910 (3.005)	400*
		3) Blanket sewing(n=3)	420 (3.511)	900 (2.000)	800*
Pandanus weaving	Lighting (lux) (lux)	1) Pandanus clipping(n=3	450 (4.041)	905 (0.577)	400*
		2) Ironing(n=3)	660 (0.577)	855 (4.725)	400*
		3) Soaking/Drying(n=3)	657 (3.214)	860 (0.577)	400*
		4) Weaving	665 (2.081)	880 (0.577)	800*

Remark: *Ministerial Regulation, Specification of occupational safety, health and working condition management standards (heat, light and noise), date 16th February B.E.2006.

Lighting levels in the plastic weaving workplace areas which holds plastic cutting and plastic weaving machineries, before the improvement measures were emplaced, were 742 and 750 Lux respectively. After the skylight installation and window cleaning, the results showed that the lighting levels in the working areas increased to 905 and 915 Lux which was even higher than the level set by governmental standards.

Lighting levels in the blanket making areas which holds cloth cutting and blanket stuffing equipments, before the improvement measures were emplaced, were 450 and 400 Lux which were at the governmental standard level. After the improvement, the results showed that the lighting levels in the blanket making areas increased to 905 and 910 Lux.

Before the improvement, the lighting level in the blanket sewing area was 420 Lux, which was under the governmental standard level (the standard level is 800 Lux). Lighting standards vary according to factors such as the length of time required to perform the task. After appropriate lighting was installed in the blanket sewing area, the results show that the lighting level increased to 900 Lux which was higher than the governmental standard level.

The main problem of the pandanus weaving group was related to the lighting condition. The processes of pandanus weaving consist of four parts, including pandanus clipping, ironing, soaking/drying and weaving. The weaving takes the longest time in the process. The lighting levels in the work place in the pandanus clipping area, ironing area and soaking/drying area were 682, 660 and 675 lux, respectively. These lighting levels were in line with the standard level (400 lux). However, the weaving area was found to be at 665 lux (the standard level at 800 lux). After a skylight and general lighting were installed, the lighting level in the hand-weaving area was increased to 880 lux which was the acceptable level according to the guidelines.

The above improvement measures were concrete solutions that were derived from the decision making discussions shared by every participant. The results were shown in Table 4.

## Discussion

 After having implemented the participatory process, the results showed that the post-test average score of the OHS knowledge, attitude and behaviors, and the work practice improvement of informal-sector workers were significantly higher than their pre-test average scores (p < 0.05). In respect to the working condition measurements, the participatory process demonstrated its ability to improve working conditions to meet necessary standards.

**Figure 2: Measurement of thermal conditions behind a heat barrier:**
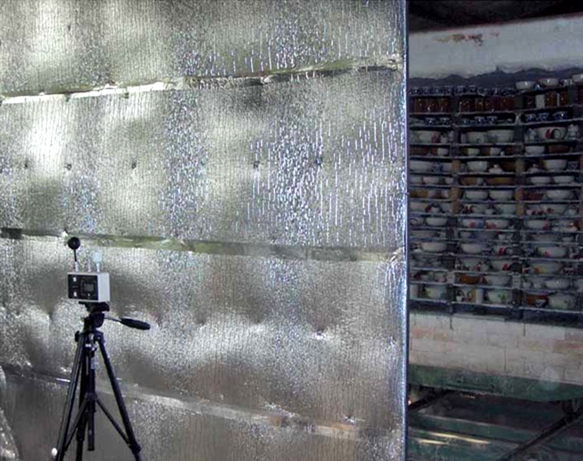


The OHS knowledge, attitude and behavior scores of the informal sector workers were increased because the workers were exposed to the capacity building component of the participatory process. The capacity building process enabled the workers to recognize risks associated with their occupation and therefore implement safety measures by using PPE and by learning about safety improvement concepts. During the participatory learning activities that were presented in this study, the informal-sector workers engaged in the group discussions and identified the OHS problems in their own sector. They subsequently attempted and accurately understood work related safety issues, and collectively proposed and implemented safety measures.

Our findings suggest that positive attitudes toward promoting safe working conditions and practices can be fostered among the informal-sector workers by raising their knowledge and skills regarding issues related to OHS through a capacity building process-the first step of the participatory process. Behavior-based safety (BBS) theory claims that positive approaches toward safe behaviors can be encouraged by offering advice, inspiration, and guidelines on how to eliminate at-risk behaviors through safety awareness.^15^ This leads to a safety culture in an organization-which is a key factor for success in safety management.

Studies by Chavalitnitkul and the National Statistics Office show that informal-sector workers lack opportunities to receive work related training and have limited knowledge when it comes to self assessment information on work related safety.^5,16^ The participatory learning techniques used in our participatory approach included using pictures and signs to facilitate the workers’ understanding of the job safety. By participating in group discussions, the workers increased their ability to improve their work condition. Additionally, the informal sector workers in our study participated in the development of the participatory process and understood the purposes of the study; therefore the workers were highly motivated to participate in the study.

In terms of the work practice improvement, our results show that the post-test average scores of the participants improved across all groups after going through the participatory process and these scores were significantly higher than the pre-test average scores. It is possible that the participatory approach helped the workers to see examples of the best practices relating to their fields. The participatory process allowed the workers to clearly understand their work related problems and to find solutions by using local wisdom. The best practices of other informal sector workers were shared during the discussion groups to make the process easier to understand. This exchange of real life experiences helped the groups in different sectors learn quickly. This outcome is similar to a study by Kawakami which indicated that the exchange of learning experience among groups helps informal sector workers clearly see examples of improvement.^7^

In terms of working conditions, the results show that the working conditions of the groups improved to reach governmental standards. A working condition improvement process using the WISE technique was implemented in the study. The WISE technique uses participatory methods to find practical and feasible improvements. Krungkraiwong et al. argue that the WISE technique can effectively reduce the risks of informal-sector workers and small-scale enterprises.^17^ Also, Kogi in his study, “Work Improvement and OSH Management Systems,” claims that the WISE technique is a methodology that can be used to find low-cost improvements.^18^ Use of the WISE technique has resulted in a reduction of occupational hazards. Informal-sector workers can determine suitable solutions according to their local ways of living.

**Figure 3: Checklist exercise and a discussion about work practice improvement:**
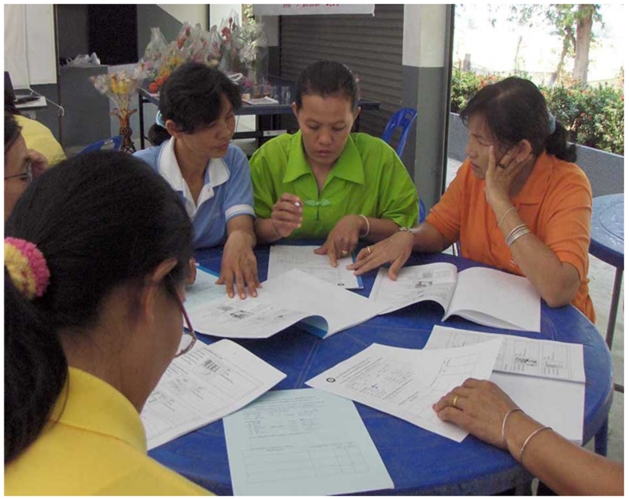


The discussions in the participatory process were usually led by a group leader. After the discussions, the participants generally agreed on how to solve the problems. For example, after the ceramic workers in the Lampang province acknowledged a lighting condition problem and heat exposure, the group leader suggested that the heat exposure should be solved before the lighting condition problem. After the discussion, all participants agreed that the heat exposure problem, due to the severity of the harm, more seriously affected the workers than the lighting condition.

In future, the participatory process should be applied to the small and medium enterprises (SMEs). The participatory process was initially designed for informal sector workers. However, the participatory process might also be used to improve the working conditions of formal-sector workers like SMEs. In formal sector groups, safety regulations and safety training are enforced by their administrators; therefore, the workers who directly face the work related risks have limited involvement in regulating safety practices. The participatory process emphasizes the participation of everyone involved. As a result, applying the participatory process could promote a formal sector group’s realization, motivation, and accountability in its safety implementation.

The findings of this study demonstrate that all steps of the participatory process (capacity building, risk analysis, problem prevention and solving, and monitoring and communication) aim at promoting the worker’s capacity to self development in safety aspects. As a result, the participatory approach could be an effective approach in promoting safety and consequently the health of the local informal sector workers in Thailand by motivating these workers to voluntarily engage in improving their own quality of life.
